# Effects of Three Different Niti Rotary Instruments on Dentin Cracks After Root Canal Preparation

**DOI:** 10.34172/joddd.42346

**Published:** 2026-06-30

**Authors:** Rhona Mariana Untari, Wignyo Hadriyanto, Rachmat Sriwijaya, Margareta Rinastiti

**Affiliations:** ^1^Resident of Conservative Dentistry Program, Faculty of Dentistry, Universitas Gadjah Mada and Bagas Waras Hospital, Klaten, Indonesia; ^2^Departement of Conservative Dentistry, Faculty of Dentistry, Universitas Gadjah Mada, Indonesia; ^3^Departement of Mechanical and Industrial Engineering, Faculty of Engineering, Universitas Gadjah Mada, Indonesia

**Keywords:** Dentin crack, Instrument design, Rotary instrument, Rotational speed

## Abstract

**Introduction:**

This study aimed to examine the effects of three distinct nickel–titanium (NiTi) rotary instruments on dentin crack formation during root canal preparation in various root segments.

**Methods:**

Thirty premolars with a single straight root canal were prepared using three rotary instruments, namely, ProTaper Next, ProTaper Gold, and HyFlex Controlled Memory (CM).

**Results:**

The ProTaper Next produced the most dentin cracks, whereas HyFlex CM produced the fewest. Most cracks occurred in the coronal third of the root compared with the middle third. Dentin crack formation is a significant problem in root canal treatment and can lead to treatment failure. Therefore, an appropriate rotary instrument and technique for root canal preparation must be selected to minimize the risk of dentin crack formation.

**Conclusion:**

The results can guide clinicians in selecting the most suitable rotary instrument for successful endodontic treatment. In addition, irrigating with 2.5% NaOCl and saline for 1 min after preparation could help reduce the incidence of dentin cracks. Further research can explore new methods to minimize the risk of dentin crack formation during root canal preparation and improve the success rate of endodontic treatment.

## Introduction

 Biomechanical instrumentation is crucial in determining the outcomes of root canal treatment. Nickel–titanium (NiTi) rotary devices are preferred for root canal preparation because of their superior consistency and efficiency over manual instruments.^1,2^ However, the rotary device might rotate more in the root canal and cause friction between the walls and the instrument and dentin defects.^3^ During root canal preparation, the instrument’s contact with the canal walls causes internal pressure and torsional reactions to the root canal walls.^4^

 During root canal preparation, a NiTi rotary device with active cutting edges and large taper forces on the root canal dentin affects stress concentrations along the dentin wall, which can create cracks, weaken the root structure, contribute to root fractures, and affect tooth prognosis after root canal therapy.^5,6^ Most dentin cracks are in the apical and middle thirds of the root canal, which result from significant pressure exerted below the cementoenamel junction (CEJ).^7^ In addition, many cracks were observed at a site 2 mm from the apex, due to the thinner dentin wall. The lateral force during instrumentation augments the probability of apical dentinal defects.^8^

 Recently, advancements have been made in thermomechanical technology and in instrument manufacturing techniques to create controlled memory (CM) wires, memory wires (M wire), and electrical discharge-machined components. These improvements aim to optimize the mechanical and microstructural features of NiTi alloy.^9^ A rotary instrument that uses M wire is ProTaper Next (PTN), whereas ProTaper Gold (PTG) and HyFlex CM (HFC) are made from CM wire.^10,11^ PTG features a convex triangle cross-section that maintains symmetrical rotation as the file advances into the root canal and keeps the file in contact with the canal wall at three points while turning, minimizing contact between the file and the root canal dentin. PTN was introduced as a file system with an asymmetrical rectangular cross-section and two contact points on the root canal walls during rotation. Both files have a progressive taper size and are used with a rotational speed of 300 rpm and torque of 2.0–5.2 Ncm.^12,11^ HFC has high flexibility and may respond to pressure, torsion, and resistance by maintaining a longer pitch. As a result, the device is less likely to become lodged in the root canal and breakage is less likely. Uniquely, it responds to pressure, torsion, and resistance by prolonging the pitch to avoid binding in the root canal and increasing the resistance to instrument fracture. HFC is characterized by its ability to recover to its original shape after being heated in an autoclave, and HC can return to its original shape after heat sterilizing. It rotates at 500 rpm with a torque of 2.5 Ncm.^13^

 Latest-generation NiTi rotary instruments have various designs; however, clinic and laboratory research are still needed to determine the efficacy of the root canal preparation, manner of preparation, and cutting efficiency, including its effect on the root canal wall, specifically cracking within the root canal that may reduce tooth resistance after root canal treatment. This study aimed to determine the influence of geometric design, rotational speed, and NiTi rotary instrument material on the initiation of dentin cracks in teeth after root canal preparation in the coronal, middle, and apical thirds.

## Methods

 The research protocol was approved by the Ethics Committee of the Faculty of Dentistry, Universitas Gadjah Mada (00453/KKEP/FKG UGM/EC/2020). Premolar teeth were used. Radiographs were taken to identify single and straight root canals, closed apex, caries, calcification, and anatomical variations of cracks in teeth.

 Thirty premolars were decoronated, leaving a length of 12 mm from the apex. The root canals were negotiated using a #15 K-file. The specimens were randomly assigned to three groups (n = 10). In group 1, an instrument consisting of an M wire with a rectangular cross-section and a variable taper, operated at 300 rpm (PTN), was used. In group 2, an instrument made from CM wire with a rotational speed of 300 rpm, a convex triangular cross-section structure, and a progressive taper size (PTG) was used. In group 3, an instrument made from CM wire with a rotational speed of 500 rpm, a triangular–square combined cross-sectional design, and a constant taper (HFC) was used. Root canal preparation was performed until the apical foramen diameter was #25 with “in-and-out movement” (pecking motion) for 1 min for each file. At each turn of the instrument, the root canal was cleaned with 2.5% NaOCl solution, then rinsed with saline.

 After preparation, the entire specimen was measured and marked for horizontal cutting into three sections using Isomet. The coronal part of each segment was marked and then stained with 2% methylene blue for 1 min (Figure 1). After drying, the specimens were observed under a stereomicroscope with × 25 and × 40 magnifications. Cracks were identified as a line extending from the root canal wall to the dentin without reaching the outer surface (incomplete crack). The data were recorded as cracks (1 point) or no cracks (0 points).^14^ The cracks were observed from three parts, namely, coronal, middle, and apical, and research data were analyzed using IBM SPSS Statistics version 23 (IBM Corp., Armonk, NY, USA) with chi-squared test and a significance level of *P* < 0.05.

**Figure 1:**
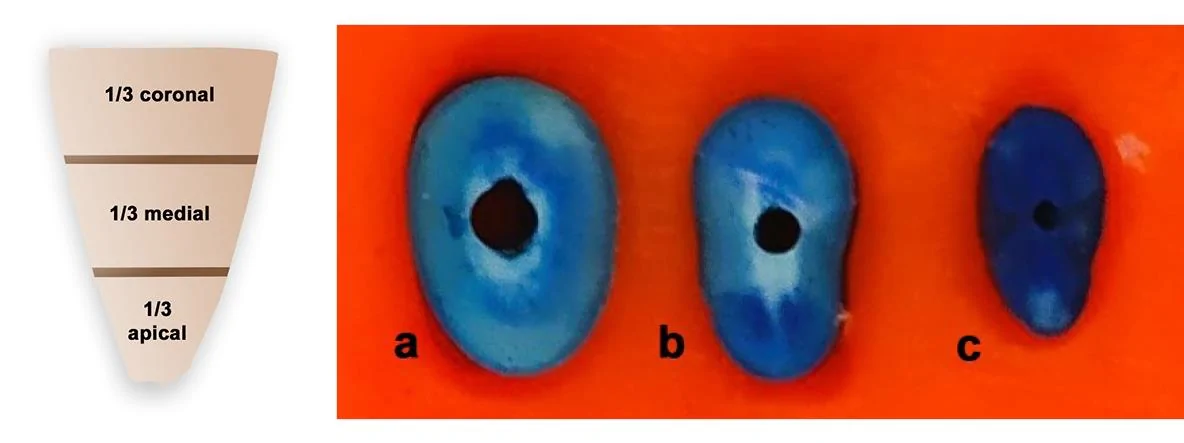
Schematic representation of a specimen sectioned horizontally at equal lengths, and staining of a) coronal, b) medial, and c) apical sections of the root.

## Results

 The chi-squared test yielded a P-value of 0.010 (*P* < 0. 05), which means differences in the dentin crack formation by the three NiTi rotary instruments with dissimilar designs used for root canal preparation (Table 1). The group prepared with an instrument that had a constant taper and triangular–square cross-section showed the least number of dentin cracks (group 3), and the group prepared with an instrument that had a variable taper and rectangular cross-section had the highest number of dentin cracks in the root canal wall (group 1).

**Table 1:** Differences in dentin crack formation between the three treatment groups with three different instrument designs

**Group**	**Dentin cracks**				
	**No**		**Yes**		***P*** **-value**
	**N**	**%**	**N**	**%**	
ProTaper Next	18	60.0%	12	40.0%	
ProTaper Gold	22	73.3%	8	26.7%	0.010*
HyFlex CM	28	93.3%	2	6.7%	

 Based on the location of the dentin fracture, a significant difference was found between the three treatment groups with different instrument designs in the coronal and apical thirds (Table 2). Most dentin cracks were found in the coronal third, whereas the least ones were in the middle third of the root canal wall (Figure 2). Table 3 shows differences in dentin crack formation across material types and rotational speeds.

**Table 2:** Differences in dentin cracks in the apical, middle, and coronal third among the three groups using different designs of rotary instruments

		**Dentin cracks**				***P*** **-value**
		**No**		**Yes**		
		**N**	**%**	**N**	**%**	
Coronal	ProTaper Next	2	20%	8	80%	0.024*
	ProTaper Gold	6	60%	4	40%	
	HyFlex CM	8	80%	2	20%	
Medial	ProTaper Next	10	100%	0	0%	0.117
	ProTaper Gold	8	80%	2	20%	
	HyFlex CM	10	100%	0	0%	
Apical	ProTaper Next	6	60%	4	40%	0.038*
	ProTaper Gold	8	80%	2	20%	
	HyFlex CM	10	100%	0	0%	

* statistically significant

**Figure 2:**
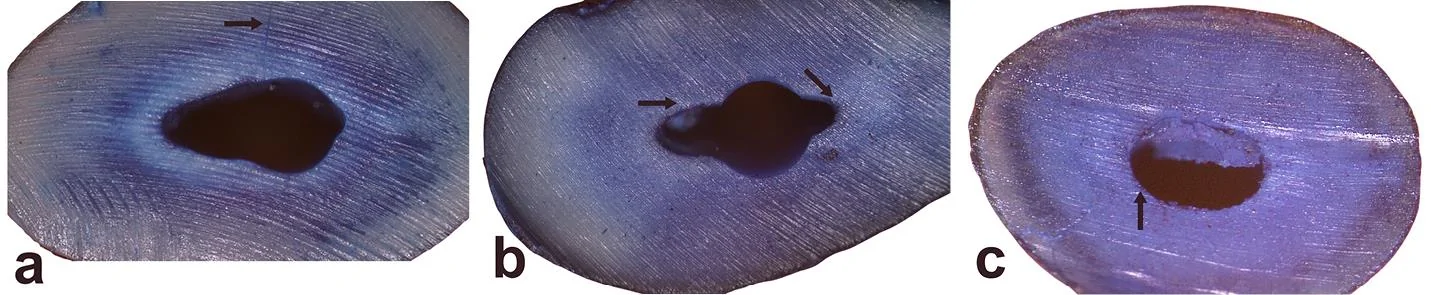
A stereomicroscopic image of three segments: a) coronal third, b) middle third, and c) apical third. The arrow indicated a crack line

**Table 3:** Differences in dentin cracks based on the types of materials and the rotational speeds of the NiTi rotary instruments

		**Dentin cracks**				***P*** **-value**
		**No**		**Yes**		
		**n**	**%**	**n**	**%**	
Rotational speed	P. Next + P. Gold (300 rpm)	40	66.7%	20	33.3%	0.006 *
	HyFlex CM (500 rpm)	28	93.3%	2	6.7%	
Types of materials	P. Next (M Wire)	18	60.0%	12	40.0%	0.015 *
	P. Gold + HyFlex CM (CM Wire)	50	83.3%	10	16.7%	

* statistically significant

 The chi-squared test revealed a statistically significant difference in postoperative root canal crack formation between the rotary instrument with a rotational speed of 300 rpm (PTN and PTG) in comparison with the instrument with a rotational speed of 500 rpm (HFC) (*P* = 0.006). Furthermore, a significant difference in the occurrence of cracks after root canal preparation was found between the M wire (PTN) and the CM wire (PTG and HFC) (*P* = 0.015).

 The instrument made of M wire, operating at 300 rpm, produced the most dentin cracks. In contrast, the instrument made of CM wire at 500 rpm produced the fewest cracks. These results demonstrate the importance of selecting the appropriate rotary instrument and technique for successful root canal treatment and highlight the need to consider factors such as material and rotational speed to minimize the risk of dentin cracks. The finding offers significant advice for clinicians in selecting the most suitable instrument and technique for successful endodontic treatment.

## Discussion

 Several studies have reported dentin damage of the root canal wall after treatment using NiTi rotary instruments. The risk of tooth fracture during endodontic treatment may be influenced by factors such as tooth type, root canal wall thickness, root canal wall diameter, cross-sectional form, instruments, and preparation technique.^15^ According to Yoldas et al.^16^ (2012), the tip configuration, cross-sectional geometry, varying or constant pitch and taper, shape of the taper, and flute of the rotary instrument contribute to cracks in the root canal dentin.

 The study results indicated a notable difference in dentin cracks between teeth prepared with three instruments of varying designs. The teeth prepared using instruments with a rectangular cross-section and variable taper, and those with a convex triangular cross-section and progressive taper size, generated more dentin cracks than teeth prepared using instruments with a constant taper and triangular–square combined cross-section (*P* = 0.010). These findings are likely due to the differences in cross-sectional shapes and taper sizes between the instruments. PTN features a rectangular cross-section and a variable taper; PTG has a convex triangular cross-section and a progressive taper; and HFC has a triangular–square combined cross-section with a constant taper.^12^

 Mechanical instrumentation with a large taper can remove more dentin and exert greater pressure on the root canal walls, potentially increasing the risk of fracture.^17^ Thus, the appropriate rotary instrument for endodontic treatment must be selected to minimize the risk of dentin cracks and tooth fractures. During mechanical instrumentation, clinicians should consider the instrument’s design, cross-sectional shape, and taper size to ensure successful root canal treatment.

 A study reported that instruments with a convex triangular cross-section distribute pressure more evenly along the root canal and have lower stress concentrations than those with rectangular cross-sections.^5^ However, in a study comparing the cutting ability and efficiency of instruments with a convex triangular cross-section with those with a rectangular cross-section on dentin cracks, Kataia^18^ (2016) found no difference between the two instruments in terms of the number of cracks on the teeth. The present study showed no significant difference in the occurrence of cracks across the root canal walls prepared with a convex triangular cross-section and those prepared with a rectangular cross-section (*P* = 0.273). This indicates that both instruments can be used to prepare root canals without causing a significant difference in the number of dentin cracks. However, when selecting the appropriate instrument for endodontic treatment, clinicians should consider other factors, such as taper size, rotational speed, and material.

 The helix angle of rotary instruments can also affect the cutting ability and effectiveness of the preparation. Instruments with a rectangular cross-section are characterized by a helix angle of 18.5°. When compared, instruments with a convex triangular cross-section have a helix angle of 19.1°, which is 10.8° higher than that of instruments with a combined rectangular cross-section, which have a helix angle of 8.3°.^19^ A higher helix angle leads to the removal of more dentin and increases the risk of dentin crack formation. Consistent with these findings, the results of this study demonstrated that teeth prepared using an instrument with a rectangular cross-section and a helix angle of 18.5° had a significantly higher number of dentin cracks (40%) than teeth prepared using an instrument with a triangular–square combined cross-section and a helix angle of 8.3° (6.7%) (P < 0.002). Therefore, clinicians should consider the helix angle of the instrument when selecting the appropriate rotary instrument for endodontic treatment to minimize the risk of dentin crack formation.

 The materials of the instrument affect the flexibility of the file. File stiffness can also cause large stress concentrations and is influenced by factors such as instrument size, taper, cross-section, technique, and material. The higher the file stiffness, the greater the pressure against the canal wall; thus, contact between the instrument and the canal wall can cause damage to the dentin.^20^ File stiffness generates higher stress concentration, increasing the risk of dentin defects that can lead to root cracks.^5^ File stiffness is associated with a cross-sectional shape, size, taper, and material of the instrument. Instrument flexibility can be affected by the design and dimensions of the instrument, chemical composition of the alloy, and thermomechanical process.^21^ Instruments with a rectangular cross-section are made of M wire, which contains 56% nickel and has a shape memory, so they can easily return to their original shape, whereas instruments with a convex triangular cross-section and those with a square–triangular combined cross-section are made of CM wire, which contains 52% nickel and have a controlle memory, so they follow the shape of the root canal. Instruments made from CM wire are more flexible than those made from M wire, so they exert less pressure on the root canal walls. The difference in the instrument material composition affects their flexibility during root canal preparation.^11,13^ In this study, instruments made from CM wire produced fewer dentin cracks than those made from M wire.

 In addition to instrument flexibility, an instrument’s cutting ability and efficiency also affect dentin crack formation. The cutting ability of the instrument is also influenced by the instrument design, the hardness and sharpness of the cutting blade, the instrument tip design, lubrication during preparation, rotational speed, and the method used. The greater the instrument’s cutting ability, the lower the probability of dentin crack formation.^18^ According to previous study, a higher file rotational speed generates less force and torque. Instruments with enhanced cutting efficacy exhibit reduced torque and shorter root canal preparation duration, minimizing the contact time between the instrument and the root canal wall^22^. The investigations used the manufacturer-recommended rotational speed and torque. The instrument operating at 500 rpm exhibited the fewest dentin cracks compared with the other two instruments operating at 300 rpm (6.67%). This shows that the cutting effectiveness of instruments at 500 rpm is greater than that at 300 rpm; according to Peter et al.^23^ (2014), an increase in rotational speed is associated with greater cutting effectiveness.

 Dane et al. ^24^ (2016) investigated the effect of torque on the canal wall and found that the formation of canals with high torque settings resulted in more dentin cracks than those with low torque settings. Higher torque settings increase pressure; thus, more pressure is transferred to the dentin, increasing the likelihood of dentin crack formation. Reducing torsional stress can increase instrument resistance and reduce the risk of dentin fracture.^25,26^ In this study, the torque setting was based on factory settings ranging from 2 to 3 N/cm^2^, except for instruments with a convex triangular cross-section, which had variable torque for each file. To determine the effect of torsion on dentin crack formation, further studies comparing the effects of each instrument are necessary.

 The shape of the root canals also affects the formation of dentin cracks. Rounded root canals show a lower and even pressure distribution than oval canals, where the cervical and middle thirds have higher fracture risks.^14^ A higher torque is generated in the apical third, and the torque increases proportionally when the instrument enters the root canal. However, in some cases, the torsion at the coronal site is higher than that in the middle, increasing the risk for coronal dentin cracks.

 Versluis et al.^8^ (2006) revealed that the middle and coronal thirds were exposed to three times greater pressure than the apical thirds. Garg et al.^3^ (2017) compared dentin fractures in three parts of the root canal and revealed that the number of teeth with cracks in the coronal third was three times more than that in the apical third. Ustun et al.^27^ (2015) also showed more dentin cracks in the coronal third of the root canal. Adorno et al.^28^ (2011) and Liu et al.^29^ (2013) also reported that the number of dentin cracks in the coronal third was higher than that in the apical third because of increased pressure in the coronal third caused by a larger taper in the coronal direction.^14^ The results of the present study align with those of previous studies, indicating a greater incidence of dentin cracks in the coronal third (46.67%) than in the middle (6.67%) and apical (20%) thirds.

## Conclusions

 The results of this study indicated that the rotary instrument’s design, material, and speed significantly affected dentin crack formation during root canal preparation. The instrument made of M wire, with a rectangular cross-section, variable taper, and rotational speed of 300 rpm, caused numerous dentin cracks. In contrast, the instrument made of CMWire material, with a triangular–square combined cross-section, constant taper, and rotational speed of 500 rpm, resulted in the fewest dentin cracks. In addition, the coronal third of the root had the highest number of dentin cracks, whereas the middle third had the fewest. These findings highlight the importance of selecting the appropriate rotary instrument and technique for successful root canal treatment and can guide clinicians in minimizing the risk of dentin crack formation. Furthermore, irrigating the canal with 2.5% NaOCl and saline after preparation could help reduce the incidence of dentin crack formation.

## Competing Interests

 The authors declare that they have no conflicts of interest.

## Data Availability Statement

 The data presented in this study are available on request from the corresponding author.

## Ethical Approval

 This study was approved by the Research Ethics Committee, Faculty of Dentistry, Universitas Gadjah Mada (No.00454/KKEP/FKG-UGM/EC/2020).

## Informed Consent

 Not applicable.
